# Effect of a Gender-Tailored eHealth Weight Loss Program on the Depressive Symptoms of Overweight and Obese Men: Pre-Post Study

**DOI:** 10.2196/mental.8920

**Published:** 2018-01-09

**Authors:** Myles D Young, Philip J Morgan

**Affiliations:** ^1^ Priority Research Centre in Physical Activity and Nutrition University of Newcastle Callaghan Australia

**Keywords:** male, weight loss, depression, behavior change, obesity, gender-sensitive

## Abstract

**Background:**

Obesity and depression are of two of the largest contributors to the global burden of disease in men. Although lifestyle behavior change programs can improve participants’ weight and depressive symptoms, the evidence is limited by a lack of male participants and a reliance on face-to-face treatment approaches, which are not accessible or appealing for many men.

**Objective:**

This study examined the effect of a gender-tailored electronic health (eHealth) program on the depressive symptoms of a community sample of overweight and obese men with or without depression. A secondary aim was to determine whether the eHealth, self-directed format of the program was a feasible and acceptable treatment approach for the subgroup of men with depression at baseline.

**Methods:**

In total, 209 overweight/obese men from the Hunter Region of Australia were assessed before and after completing a self-administered eHealth weight loss program over 3 months. To increase engagement, most program elements were socio-culturally targeted to appeal specifically to men and included printed materials, a DVD, motivational text messages, online- or app-based self-monitoring, and other weight loss tools (eg, pedometer). Depressive symptoms were measured with the validated 8-item Patient Health Questionnaire (PHQ-8). Program feasibility and acceptability were assessed with a process questionnaire plus recruitment and retention rates. Changes in depressive symptoms and weight were examined using intention-to-treat linear mixed models, adjusted for the centered baseline score and other covariates. Effect sizes were estimated with Cohen’s d.

**Results:**

At baseline, the mean weight and age of the sample was 105.7 kg (standard deviation [SD] 14.0) and 46.6 years (SD 11.3), respectively. Overall, 36 men (36/209, 17.2%) were experiencing depression (PHQ-8 score ≥10). Retention rates were comparable between men with and without depression (32/36, 88.9% vs 145/173, 83.8%; *P*=.44). At posttest, depressive symptoms had reduced by 1.8 units (95% CI 1.3 to 2.3; *P*<.001; d=0.5) for the whole sample. These improvements were particularly notable in the subgroup of men with depression (-5.5 units; *P*<.001; d=1.0) and 72.2% (26/36) of this subgroup no longer met the criterion for depression at posttest. A corresponding, albeit smaller, intervention effect on depressive symptoms was also observed in men without depression (-1.0 units; *P*<.001; d=0.4). The overall intervention effect on weight was -4.7 kg (d=1.3), which did not vary significantly by depression status. Program acceptability, feasibility, and online engagement metrics were also comparable between men with and without depression.

**Conclusions:**

A gender-tailored eHealth lifestyle program generated short-term improvements in the mental health of overweight and obese men, particularly for men with depression at baseline. Despite receiving no personalized support, men with depression reported high levels of satisfaction and engagement with the program. As such, a longer-term controlled trial testing an adapted version of the program for this subgroup is warranted.

**Trial Registration:**

Australian New Zealand Clinical Trials Registry: ACTRN12612000749808; https://www.anzctr.org.au/ Trial/Registration/TrialReview.aspx?id=362575 (Archived by WebCite at http://www.webcitation.org/6wJvbRsNW)

## Introduction

Obesity and major depressive disorder, henceforth referred to as depression, are two of the largest global health concerns in men [1,2]. In addition, the two conditions are reciprocally associated [3], with the presence of obesity increasing the risk of depression, and vice versa. In Australia, 70.1% of men are overweight/obese [4] and up to 20% of these men may also be experiencing depression [5]. Although both conditions also affect women, men are much less likely to seek help [6,7], and men also experience unique health consequences. For example, men are more likely to store excess fat abdominally, which increases their risk of chronic disease [8], and are three times more likely to die by suicide [9]. As such, effective and scalable programs are urgently needed to reduce rates of obesity and depression in men.

Encouragingly, behavior change programs have shown initial efficacy to generate clinically meaningful improvements in participants’ body weight [10] and depressive symptoms [11]. However, the evidence-base is undermined by a lack of men, who represent 27% of participants in behavioral weight loss trials [12] and 20% in trials where depression is a key outcome [11]. Many previous programs have also included multiple in-person consultations, which reduce program accessibility and appeal for many men [7,13].

The *Self-Help, Exercise and Diet using Information Technology* (SHED-IT) program is an electronic health (eHealth) weight loss intervention that is socio-culturally tailored to appeal specifically to men [14-16]. While substantially less intensive than previous programs [6,17], the program has assisted men to achieve clinically meaningful improvements in weight and multiple health behaviors [15]. However, the impact of the program on men’s mental health has not been well established. Although the program has been tested in an efficacy trial (SHED-IT Weight Loss RCT [14]) and an effectiveness trial (SHED-IT Community RCT [15]), these studies did not assess men’s depressive symptoms. In addition, while eHealth programs have been flagged as a promising treatment for men with depression [18], little research has examined men’s perceptions of these programs; particularly those relating to lifestyle modification.

Thus, the primary aim of the current study was to examine the effect of the SHED-IT program on the depressive symptoms of a community sample of overweight and obese men with or without depression. A secondary aim was to determine whether program engagement and satisfaction metrics were comparable between men with and without depression.

## Methods

### Study Design

The data for this investigation were sourced from the SHED-IT weight loss maintenance trial, which has been published elsewhere [19,20]. Briefly, the study included 209 overweight/obese men from the Hunter Region of Australia who were recruited via a university media release [20]. Eligibility criteria were: (1) age 18-65 years old, (2) body mass index 25-40 kg/m^2^, (3) Internet and mobile phone access, and (4) <5% weight loss in previous 6 months. The current study reports data from the initial weight loss phase of the trial (pre-post design), in which 209 men received the SHED-IT Weight Loss Program for 3 months before being randomized into one of two weight loss maintenance conditions. The study received ethics approval from the University of Newcastle’s Human Research Ethics Committee and was prospectively registered (ACTRN12612000749808).

### The SHED-IT Program

All men received the SHED-IT program, which was a self-administered eHealth program that included no personalized intervention components. The program consisted of: (1) the *SHED-IT Weight Loss Handbook* and *Weight Loss Log Book for Men*, (2) the *SHED-IT Weight Loss DVD for Men*, (3) self-monitoring tools (ie, tape measure, pedometer), and (4) weekly motivational text messages (standardized). During the program men were advised to self-monitor their physical activity and diet using the freely available CalorieKing Australia website [21] or MyFitnessPal app [22] to create a 2000 kilojoule energy deficit on most days. After receiving the resource pack and an instruction sheet, participants were not provided with additional support during the intervention period.

To increase engagement, most elements were socio-culturally targeted for men, with attention given to both surface-structure components (eg, male-specific pictures and research findings) and deep-structure, value-based components (eg, humor, frank and realistic communication). The program taught men how to lose weight sustainably, without eliminating valued discretionary choices (eg, beer). Extensive details on program development and components are provided elsewhere [20].

### Measures

Depressive symptoms were measured with the validated 8-item Patient Health Questionnaire (PHQ-8) [23], with men indicating how often in the past two weeks they experienced a range of symptoms associated with major depression (range=0-24). Following established guidelines [24], PHQ-8 scores ≥10 were used to indicate the presence of depression. In a validation study, 88% of people with major depression reported a PHQ-8 score >10 and 88% of people without major depression reported a PHQ-8 score <10 [23]. Weight was measured without shoes on a digital scale to 0.01 kg. In addition to recruitment and retention rates, program satisfaction metrics were collected using a revised process evaluation questionnaire, which was originally developed for use in a previous study [15]. In the current trial, the questionnaire also included new questions relating to engagement with online program components, which were developed specifically for this study.

### Analyses

All analyses were conducted in IBM SPSS Statistics 22 (Armonk, NY; IBM Corp). Changes in depressive symptoms and weight were examined using linear mixed models, and adjusted for the centered baseline score and the following covariates: age, socio-economic status, physical activity (steps/day), energy intake (kilojoules/day), and risky alcohol consumption. Linear mixed models are consistent with an intention-to-treat approach as they model missing responses with a likelihood-based analysis that includes all available data. Effect sizes were represented with Cohen’s d (mean change/standard deviation [SD] of change). Differences between men with and without depression for categorical outcomes were assessed with Chi-square tests.

## Results

### Baseline Characteristics and Participant Flow

As reported elsewhere [19], 209 of 236 men who met the study’s eligibility criteria were enrolled after returning consent. The mean weight and age of the sample were 105.7 kg (SD 14.0) and 46.6 years (SD 11.3), respectively. Overall, 36 men (36/209, 17.2%) reported comorbid depression at baseline. Although no incentives/reimbursements were offered for attending assessments, the posttest retention rate was 84.7% (177/209). A greater proportion of men with depression attended the posttest assessment (32/36, 88.9%) compared to those without (145/173, 83.8%), but the difference was not significant (χ^2^=0.6; degrees of freedom=1; *P*=.44).

**Figure 1:**
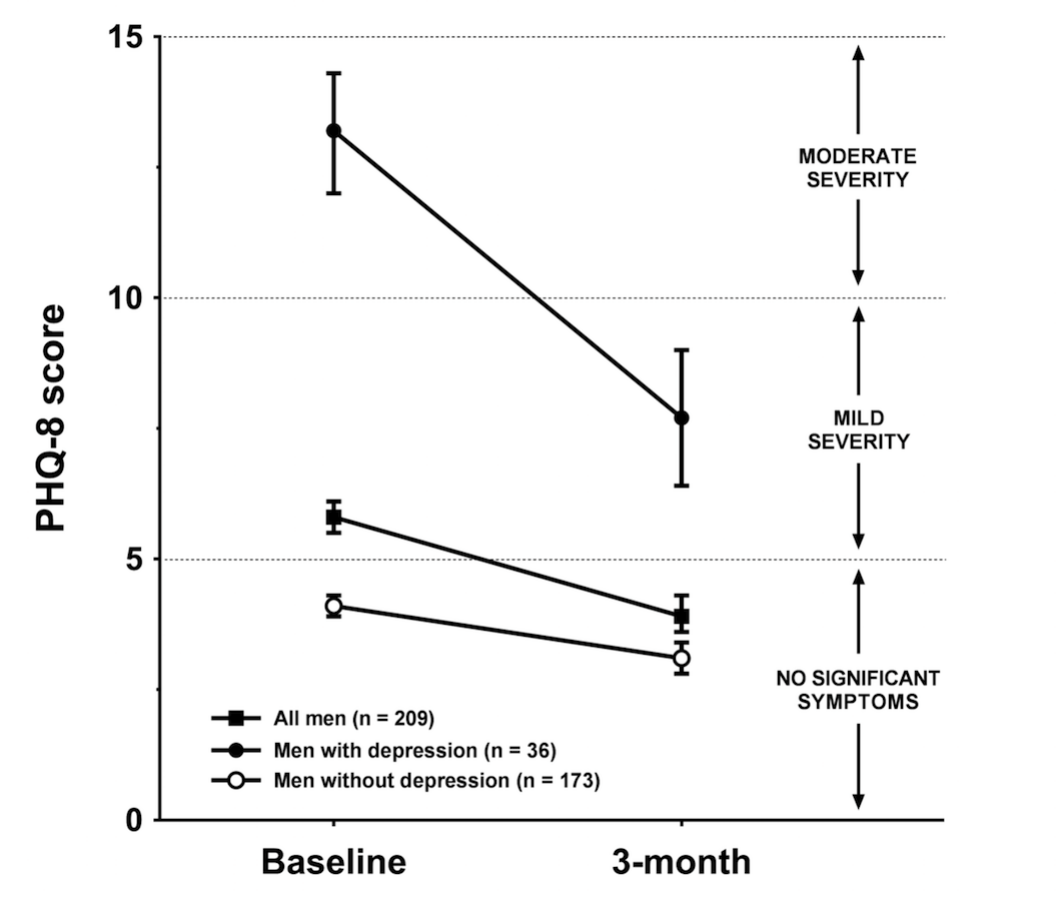
Intention-to-treat analysis of changes in depression symptoms by baseline depression status for overweight and obese men in the SHED-IT trial.

### Program Effect on Depressive Symptoms and Weight

As seen in Figure 1, men with depression reported a substantial decrease in depressive symptoms during the study (adjusted mean difference: -5.5 units, 95% CI -7.2 to -3.8; d=1.0). Consequently, 72.2% (26/36) of these men no longer met the criterion for depression at posttest. A corresponding, albeit smaller, intervention effect on depressive symptoms was also observed in men without depression at baseline (adjusted mean difference: -1.0 units, 95% CI -1.4 to -0.6; d=0.4). Overall, these changes represented a mean decrease in depressive symptoms of 1.8 units (95% CI 1.3 to 2.3; d=0.5) for the sample. The overall intervention effect on weight was -4.7 kg (95% CI -5.2 to -4.2; d=1.3), which did not vary significantly by depression status.

### Program Acceptability and Online Engagement

No significant differences were detected between men with and without baseline depression for the program acceptability questions included in the posttest process evaluation (Table 1). Overall, 82.7% (24/29) of the men with depression who completed the process evaluation reported that the program provided them with sufficient support to lose weight, and 93.1% (27/29) indicated they would recommend the program to their friends. The men also reported high levels of agreement that the program resources were enjoyable to read (23/29, 79.3%) and watch (24/29, 82.7%).

Usage rates for the online program components were comparable between men with and without depression (Table 2). Of the 82.8% (24/29) of men with depression who accessed the online components, the median self-reported usage rates were 4 x 10-minute visits/week for the CalorieKing website and 3 x 5-minute visits/week for the MyFitnessPal app, which aligned with program recommendations.

**Table 1:** Program satisfaction indicators from men with and without depression at baseline.

Process evaluation questions		Agree/Strongly Agree, n (%)			*P* value
		Men with depression (n=29)	Men without depression (n=126)	Total^a^ (N=155)	
**SHED-IT program**					
	The SHED-IT program provided me with the support I needed to lose weight	24 (82.8)	114 (90.5)	138 (89.0)	.23
	The SHED-IT program corrected some wrong beliefs I had about physical activity, nutrition, and weight loss	23 (79.3)	88 (69.8)	111 (71.6)	.31
	I now have a much better understanding of energy balance and weight loss	27 (93.1)	110 (87.3)	137 (88.4)	.38
	I would recommend SHED-IT to my friends	27 (93.1)	119 (94.4)	146 (94.2)	.78
**CalorieKing website**					
	The website was easy to understand	20 (69.0)	81 (64.3)	101 (65.2)	.93
	Recording my daily food and exercise on the website was time consuming	17 (58.6)	61 (48.4)	78 (50.3)	.50
	The website was a valuable tool to help me understand how to lose weight	17 (58.6)	78 (61.9)	95 (61.3)	.45
**Other resources**					
	The Weight Loss Handbook for Blokes was enjoyable to read	23 (79.3)	87 (69.0)	110 (71.0)	.41
	The Weight Loss DVD for Blokes was enjoyable to watch	24 (82.8)	86 (68.3)	110 (71.0)	.22
	The DVD helped me to understand the weight loss fundamentals	24 (82.8)	91 (72.2)	115 (74.2)	.43

^a^Total number of participants who attended the posttest assessment and completed the process evaluation (155/209, 74.2% of baseline sample).

**Table 2:** Men’s engagement with the online components of the SHED-IT weight loss program.

Online engagement indicators		Men with depression (n=29)	Men without depression (n=126)	Total (N=155)
**Online engagement, n (%)**				
	Accessed CalorieKing website	21 (72.4)	96 (76.2)	117 (75.5)
	Accessed MyFitnessPal app	7 (24.1)	22 (17.5)	29 (18.7)
	Accessed website or app	24 (82.8)	103 (81.7)	127 (81.9)
**CalorieKing usage**^a^**, median (inter-quartile range)**				
	Visits/week	4 (7)	3 (4)	3 (4)
	Duration/visit (minutes)	10 (20)	10 (10)	10 (10)
**MyFitnessPal usage**^a^**, median (inter-quartile range)**				
	Visits/week	3 (6)	3 (14)	3 (10)
	Duration/visit (minutes)	5 (12)	5 (5)	5 (6)

^a^Self-report estimate from participants who accessed each online component.

## Discussion

This study revealed that an eHealth weight loss program designed specifically for men concurrently and significantly reduced men’s weight and depressive symptoms. Although a recent review determined that lifestyle modification could effectively reduce depressive symptoms [11], 80% of participants in the review were female, 58% of included studies recruited females only, almost all programs were delivered in a face-to-face group format, and no programs were socio-culturally tailored for men [11]. Thus, the current findings represent an important contribution to the literature.

Despite the self-administered nature of SHED-IT, the intervention effect on men’s depressive symptoms was comparable to those observed in more intensive interventions [11]. Furthermore, while depression is often characterized by feelings of worthlessness and apathy, men with depression in the current study reported comparable levels of program satisfaction and engagement to men without depression, despite receiving no personal support. Notably, the clinical improvements observed in this subgroup were similar to those achieved in other psychological treatments, including cognitive-behavioral therapy [25], in which participants can attend up to 16 sessions over 3-4 months. The current effect size for men with depression (d=1.0) was also comparable to effects observed in therapist-guided eHealth interventions for depression (d=0.6-1.9) and superior to previous self-guided eHealth interventions (d=0.3-0.7) [26]. In previous studies, SHED-IT participants reported medium-to-large improvements in key outcomes linked to depression in men [15], including physical activity [27] and risky alcohol consumption [28], which may partially explain these positive findings.

This study has some limitations to acknowledge. Although previous studies have established the effectiveness of the SHED-IT program over a control group [15], this pre-post evaluation was the first to assess depressive symptoms. As such, the unique impact of the program over other variables (eg, regression to the mean) could not be quantified. Furthermore, as depressive symptoms were a secondary outcome of the overall trial and the data were collected over a relatively short time frame, these results should be interpreted with caution and subject to examination in a fully-powered trial. Despite this, the study provided encouraging indicators for the potential of gender-tailored eHealth lifestyle programs to engage and improve the mental health of overweight and obese men in the short-term. Given the particular appeal and efficacy of the program for men with depression, a longer-term controlled trial testing a version of the program specifically tailored for this subgroup is warranted.

## Abbreviations

eHealth: electronic health
PHQ-8: Patient Health Questionnaire (8-item)
SD: standard deviation
SHED-IT: Self-Help, Exercise and Diet using Information Technology
